# Multi-Omics Reveals the Immunological Role and Prognostic Potential of Mitochondrial Ubiquitin Ligase MARCH5 in Human Breast Cancer

**DOI:** 10.3390/biomedicines9101329

**Published:** 2021-09-26

**Authors:** Pei-Yi Chu, Yen-Dun Tony Tzeng, Yi-Han Chiu, Hung-Yu Lin, Chen-Hsin Kuo, Ming-Feng Hou, Chia-Jung Li

**Affiliations:** 1 College of Medicine, National Chung Hsing University, Taichung 402, Taiwan; chu.peiyi@msa.hinet.net; 2 Department of Pathology, Show Chwan Memorial Hospital, Changhua 500, Taiwan; 3 School of Medicine, College of Medicine, Fu Jen Catholic University, Taipei 242, Taiwan; 4 Department of Health Food, Chung Chou University of Science and Technology, Changhua 510, Taiwan; 5 National Institute of Cancer Research, National Health Research Institutes, Tainan 704, Taiwan; 6 Department of Surgery, Kaohsiung Veterans General Hospital, Kaohsiung 813, Taiwan; seeoutony@gmail.com; 7 Institute of Biomedical Sciences, National Sun Yat-sen University, Kaohsiung 804, Taiwan; 8 Department of Microbiology, Soochow University, Taipei 111, Taiwan; chiuyiham@scu.edu.tw; 9 Research Assistant Center, Show Chwan Memorial Hospital, Changhua 500, Taiwan; linhungyu700218@gmail.com; 10 Department of Obstetrics and Gynecology, Kaohsiung Veterans General Hospital, Kaohsiung 813, Taiwan; xeriok70767@gmail.com; 11 Division of Breast Surgery, Department of Surgery, Center for Cancer Research, Kaohsiung Medical University Chung-Ho Memorial Hospital, Kaohsiung 807, Taiwan; mifeho@kmu.edu.tw; 12 Institute of BioPharmaceutical Sciences, National Sun Yat-sen University, Kaohsiung 804, Taiwan

**Keywords:** multi-omics, MARCH5, immune infiltration, prognosis

## Abstract

E3 ubiquitin-linked enzyme MARCH5, also known as membrane-associated circular finger 5, is an enzyme encoded by the human *MARCH5* gene. The main objective of this study was to visualize the prognosis of *MARCH5* in breast cancer and to determine the relationship between *MARCH5* expression and tumor immunity. MARCH5 expression was significantly higher in several cancers, including breast cancer (BRCA), compared with corresponding normal tissues. Not only was high *MARCH5* expression associated with poorer overall survival, but also *MARCH5* expression was positively correlated with the number of tumor-infiltrating immune cells in BRCA malignant tissues. Furthermore, *MARCH5* expression showed a strong correlation with various immune markers of BRCA, suggesting its role in regulating tumor immunity. MARCH5 is a useful prognostic biomarker in several cancers, and its expression is highly correlated with tumor immune cell infiltration, and increased *MARCH5* expression may serve as a new biomarker for BRCA diagnosis and prognosis.

## 1. Introduction

Breast cancer is one of the most common malignancies in women. Despite the rapid advances in the treatment of breast cancer, its incidence remains high and has tended to increase year by year in recent years, with a trend toward younger age [1]. The causes and pathogenesis of breast cancer are complex and have not been fully understood by current research. However, the occurrence and development of malignant tumors is an extremely complex and changing process, which occurs with the joint participation and interaction of oncogenes [2]. Tumorigenesis is not only a genetic lesion, but also an immune reactive lesion. When the immune function of the body is disrupted and the immune factor is abnormal, leading to the incompetence of immune surveillance, it also leads to the occurrence and development of malignant tumor [3,4].

Tumor-infiltrating immune cells play a key role in the development of cancer. Tumor-infiltrating lymphocytes infiltrate more strongly at the front of the primary tumor infiltration than the surrounding cancer deposits and are a beneficial prognostic factor for many cancers [5]. Cytotoxic T lymphocytes, which express CD8+ on the cell surface, play an important role in anti-cancer immunity. Identification of different types of immune cells in tumor microenvironment (TME) can help predict the prognosis of cancer. The survival of breast cancer cells may depend on the interaction between cancer cells and the immune cells that constitute the TME. TME plays a key role in cancer progression and response to treatment, thereby affecting patient prognosis.

MARCH family proproteins are localized to the plasma membrane and the membranes of intracellular organelles such as endosomes, endoplasmic reticulum (ER), and mitochondria. MARCH5, also known as MITOL (mitochondrial ubiquitin ligase), has four transmembrane structural domains and is present in the outer mitochondrial membrane (MOM) with the RING structural domain exposed in the cytoplasm. MARCH5 is a transmembrane protein located in the outer mitochondrial membrane and is a mitochondrial membrane-associated ring-finger E3 ubiquitin ligase. Its main function is to regulate the intracellular mitochondrial fission machinery [6], but it also appears to be involved in other processes such as mitochondrial quality control [7], mitotic phagocytosis [8], and antiviral signaling [9]. Overexpression of *MARCH5* was previously reported to lead to mitochondrial elongation. MARCH5 binds to MFN2 and DNM1L/Drp1, which are involved in mitochondrial fusion and division, respectively [10].

We explored *MARCH5* gene expression profiles by systematically analyzing the association of *MARCH5* expression with the prognosis of breast cancer patients confirmed in multiple databases. In addition, we explored the association of *MARCH5* expression with immune infiltration through the TIMER databases. Our findings demonstrate the prognostic value of MARCH5 in BRCA and provide new insights into the correlation and activity mechanisms between MARCH5 expression and tumor immunity.

## 2. Materials and Methods

### 2.1. Tissue Microarrays and Immunohistochemistry Analysis

Tissue microarray (TMA) slides (CBA4) containing human breast cancer, metastatic, and normal tissues were purchased from SuperBioChips Laboratories (Seoul, Republic of Korea). Immunohistochemistry (IHC) assays and scoring methods were performed as described. The slides were treated with anti-MARCH5 antibody (1:100, ABclonal, Boston, MA, USA). IHC analyses included a scoring system involving two aspects, namely, staining intensity and percentage of positive cells. The staining intensity was divided into four grades, including 0 (no signal), 1 (weak signal), 2 (moderate signal), and 3 (strong signal). The total score ranged from 0 to 300, calculated as staining intensity × percentage of positively labeled cells.

### 2.2. Oncomine Database

Oncomine is a large-scale oncogenic gene microarray database, covering 65 gene microarray datasets, 4700 microarrays, 715 datasets, 86,733 cancer data, and 12,764 normal tissue data [11]. This database can be used to analyze gene expression differences, find outliers, and predict co-expressed genes. The data from Oncomine can be classified based on clinical information such as tumor stage, grade, and tissue type. In our analysis, *p* < 0.05, 2-fold change and top 10% of gene classes were set as thresholds.

### 2.3. GEPIA2 Database

GEPIA2 is a web-based interactive tool (http://gepia2.cancer-pku.cn/#index, accessed on 24 August 2021) for rapid analysis and retrieval of data based on TCGA and GTEx datasets [12]. The database provides an interactive and customizable set of features including differential expression analysis, spectral mapping, correlation analysis, survival analysis, and genetic analysis. Survival results are displayed by Kaplan–Meier curves, where HR and *p* values are derived from log-rank tests.

### 2.4. Immunological Databases

The Tumor Immunology Estimation Resource (TIMER) is an interactive, user-friendly online tool for systematically assessing the expression of gene sets associated with infiltrating immune cells in TCGA data [13,14]. In this study, the association between MARCH5 expression and immune cell infiltration in breast cancer was analyzed. In addition, TIMER also investigated the association between *MARCH5* and various genetic markers of tumor-infiltrating immune cells.

### 2.5. cBioPortal Database

The cBioPortal includes a web resource of genomic data from 9555 breast cancer pathology reports from the TCGA. It provides visualization, analysis, and download of large-scale cancer genomics data from genomic profiles. *MARCH5* mutations, copy number variants (CNV), and gene co-occurrence in breast cancer are analyzed by the cBioPortal tool (https://www.cbioportal.org/, accessed on 24 August 2021) [15].

### 2.6. bc-GenExMiner Database

Breast Cancer Gene Expression Miner (bc-GenExMiner) was developed as a web-based tool to provide statistical mining tools for breast cancer transcriptome data (DNA microarray (*n* = 11,359) and RNA-seq (*n* = 4712)) [16]. This web-based application, based on DNA microarray results, is called the bc-GenExMiner and improves the performance of prognostic gene analysis by using the same bioinformatics process. The database provides clinical data and genomic information of clinical breast cancer patients.

### 2.7. Protein–Protein Interaction Network Construction and Analysis

Both databases provide the analysis of functional interactions between proteins and provides insights into the mechanisms of disease onset or development. Co-expressed genes that co-localize or interact directly or with MARCH5 targets were identified using GeneMania. The STRING (https://string-db.org/, accessed on 24 August 2021) and GeneMania databases (https://genemania.org/, accessed on 24 August 2021) analyze physical and genetic interaction data from GEO and BioGRID and infer the functional network of MARCH5 [17,18].

### 2.8. LinkedOmics Database

LinkedOmics is a publicly available portal that includes multi-component data from all 32 TCGA cancer types and 10 Clinical Proteomics Tumor Analysis Consortium (CPTAC) cancer cohorts [19]. In addition to providing mRNA or protein expression signatures of genomic alterations, candidate biomarkers of clinical attributes, candidate target genes for transcription factors, and protein kinases are presented. It also integrates and concatenates association results generated from several database modules to support multi-omics analysis in cancer type or pan-cancer analysis.

### 2.9. Statistical Analysis

Statistical methods were as previously described [20]. Correlation of gene expression was assessed using Spearman’s correlation coefficient. Statistical differences were analyzed using GraphPad Prism (GraphPad Software, La Jolla, CA, USA) by performing a *t*-test or Fisher’s exact test for both groups and a one-way ANOVA test for one group. Kaplan–Meier curves were plotted to investigate survival trends, and *p*-values were evaluated using a log-rank test. A *p*-value of less than 0.05 was considered statistically significant. Statistical significance, * *p* < 0.05; ** *p* < 0.01; *** *p* < 0.001; **** *p* < 0.0001.

## 3. Results

### 3.1. Characteristics, Mutations, and Copy Number Alterations in BRCA

**Figure 1:**
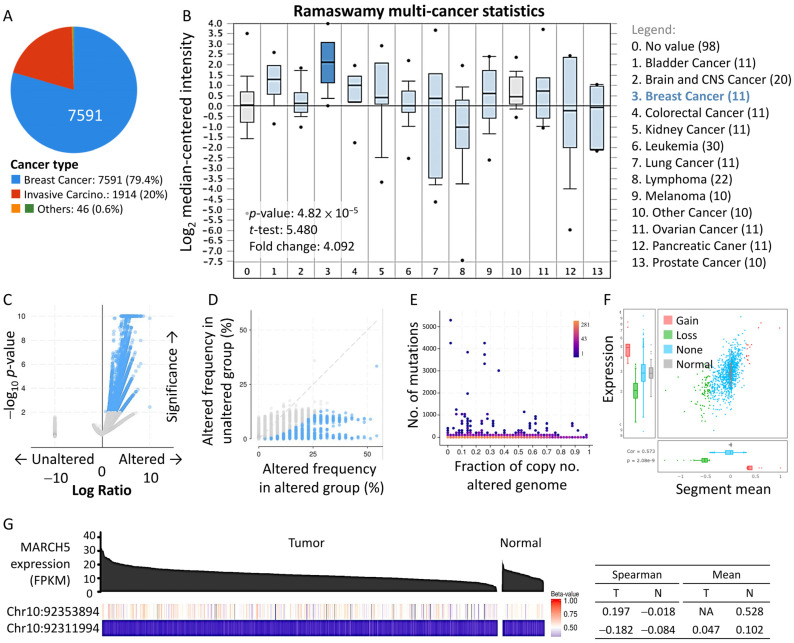
Functional enrichment analysis of *MARCH5* in BRCA demographics and clinical events. (**A**) Circle graphs show percentage of cancer type in the study. (**B**) Oncomine database analysis of the expression of *MARCH5* in different types of cancer. (**C**,**D**) Volcano and scatter plots exhibiting genes associated with mutations altered frequency in *MARCH5*. (**E**) Proportion graph indicates the ratio of mutation counts and the range of changes in the number of genome alters. (**F**) A combination of scatter plots and box plots to show a more detailed view of the distribution and correlation of CNVs in breast cancer types. (**G**) Relationship between *MARCH5* and two different datasets from LinkedOmics (http://linkedomics.org/login.php, accessed on 24 August 2021). Red lines represent a positive correlation, and blue lines represent a negative correlation.

The TCGA dataset of prostate cancer (*n* = 7597) was analyzed to identify changes in breast cancer genes and to screen for potential genes. The majority of BRCA malignancies were attributed to breast cancer (79.4%) and invasive carcinoma (20%) (Figure 1A). Using the Oncomine dataset, we found that *MARCH5* expression levels in the Ramaswamy multi-cancer dataset ranked first among cancers, with *MARCH5* levels two times higher than those in normal tissues (Figure 1B). We also analyzed the frequency of co-occurrence of gene alterations with *MARCH5* gene alterations (Figure 1C) and found a total of 10953 genes with co-occurrence of gene alterations enriched with *MARCH5* altered and unaltered cohorts. Figure 1D shows the different frequencies of alterations in co-occurring genes. Figure 1E shows and overview of clinical attributes and scatter plots of mutation counts and genomic alteration scores for each case in the TCGA breast cancer study (Figure 1E). In addition, significant changes in *MARCH5* gain and loss are observed in the CNV ratio distribution and box line plots (Figure 1F). Similarly, when we looked at *MARCH5* expression in all cancer types, we observed high levels of *MARCH5* expression in breast cancer tumor tissues compared with normal tissues at Chr10:92353894 (Figure 1G).

**Figure 2:**
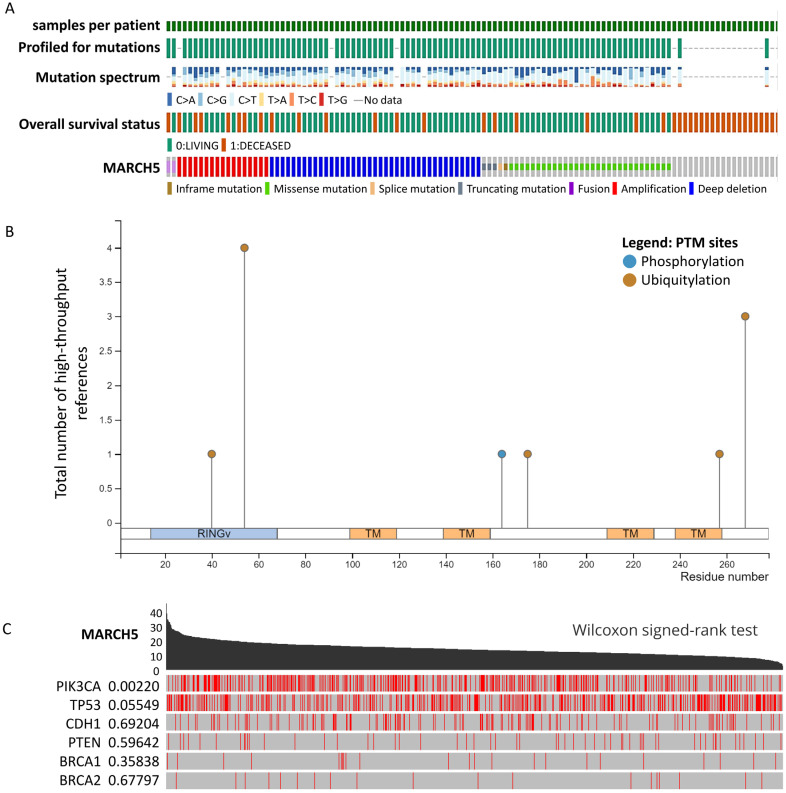
Mutation feature of *MARCH5* in different cancers in TCGA. (**A**) A visual summary on a query of genetic alteration of *MARCH5* in TCGA dataset. (**B**) The alteration frequency with mutation site is displayed. (**C**) The relationship between *MARCH5* and six highly mutated genes in breast cancer. Red line represent mutation site.

To understand the genetic changes of MARCH5, we mined that *MARCH5* gene was mutated in various cancers by querying the copy number change data and mutation percentage of various tumor samples recorded in cBioportal (Figure 2A). From the mutations occurred frequently in the RING region from the graph of *MARCH5* gene and encoded proteins (Figure 2B), we investigated the potential correlation between *MARCH5* expression in breast cancer and several mutations common to breast cancer and showed the correlation between *MARCH5* expression and each mutation, with *PIK3CA* being correlated (*p* = 0.00220) (Figure 2C).

### 3.2. Diagnostic and Prognostic Value of MARCH5 in BRCA

**Figure 3:**
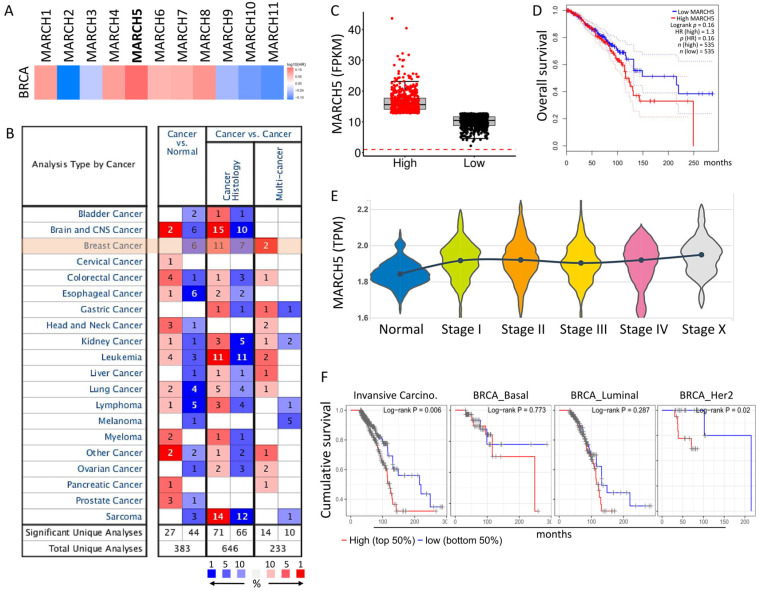
*MARCH5* expression levels in different human cancers and survival curves comparing the high and low expression in TCGA database. (**A**) GEPIA2 software analyzed the expression of *MARCH* family genes and the overall survival of breast cancer in TCGA. (**B**) The expression of *MARCH5* in different cancer tissues compared with normal tissues. The number in each cell is the number of datasets. The datasets were obtained with the following parameters: *p*-value threshold of 0.01. (**C**) Box plot showing the expression levels of the epigenetic regulatory genes in BRCA. (**D**) Overall survival analyzed using the Kaplan–Meier method with log-rank testing according to *MARCH5* expression. (**E**) Violin plot showing the expression levels of the *MARCH5* in different stages of BRCA. (**F**) Analysis of survival rates of different types of breast cancer based on TCGA database.

We used datasets from TCGA and GEPIA to investigate the relevance of MARCH family gene expression in the prognosis of breast cancer patients. Among MARCH family genes, only upregulation of *MARCH5* expression was highly associated with poor prognosis and survival (Figure 3A). Further comparison of *MARCH5* expression by Oncomine in various tumors and normal tissues also revealed high levels in breast cancer (Figure 3B). Next, we determined the transcriptional expression of the target genes differentially expressed between breast cancer and normal tissues in TCGA. It was found that the mRNA levels of *MARCH5* were significantly increased in breast cancer, and the overall survival rate of breast cancer patients with high levels of *MARCH5* expression was lower (Figure 3C,D). Subsequently, in order to better understand the relationship between *MARCH5* and clinicopathological features, we analyzed *MARCH5* at different pathological stages. The results showed that the average expression level of *MARCH5* tended to increase with the development of TNM pathological stages (Figure 3E). We classified breast cancer patients into different types, and the results showed that high levels of tumor *MARCH5* expression had a lower overall survival rate in patients with invasive breast cancer (*p* = 0.006) (Figure 3F), which also suggests that MARCH5 may have a potential role in regulating the carcinogenesis of breast cancer.

### 3.3. Overexpression of MARCH5 in Patients with Malignant Breast Cancer in Multiple Databases

**Figure 4:**
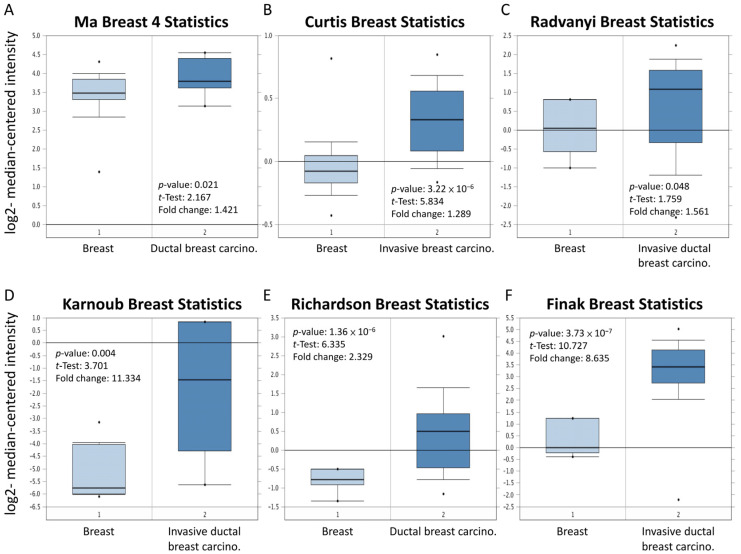
Box and whiskers plots of Oncomine data on *MARCH5*. *MARCH5* mRNA levels from (**A**) Ma Breast 4 statistics cohort, (**B**) Curtis Breast Statistics cohort, (**C**) Radvanyi Breast Statistics cohort, (**D**) Karnoub Breast Statistics cohort, (**E**) Richardson Breast Statistics cohort, and (**F**) Finak Breast Statistics cohort in BRCA and normal tissue. Note: *p* < 0.05 indicates statistical significance; MARCH5 was among the top 1% overexpressed genes in all six different datasets of BRCA.

**Figure 5:**
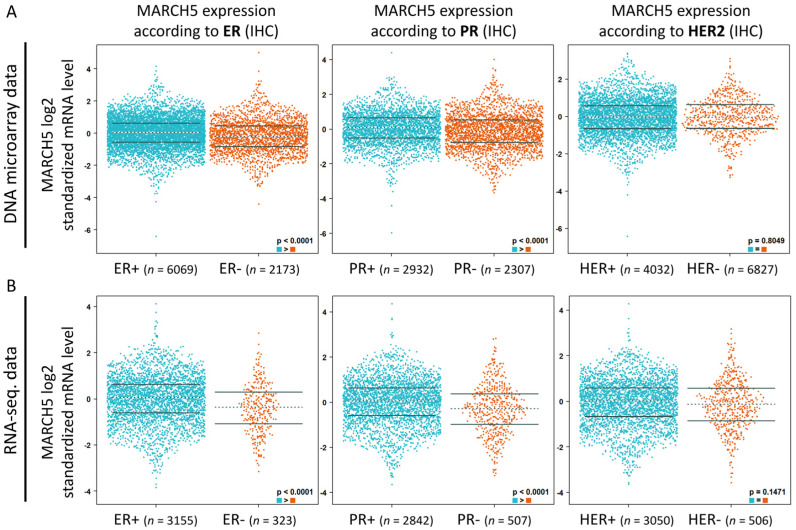
*MARCH5* transcription in BRCA (bc-GenExMiner). (**A**,**B**) *MARCH5* mRNA expression levels were shown in breast cancer patients by bee swarm in DNA microarray datasets and RNA-sequencing datasets. (ER: estrogen receptor; PR: progesterone receptor; HER2: human epidermal growth factor receptor 2).

To verify the role of MARCH5 in breast cancer, we verified the expression of *MARCH5* in different types of breast cancer using the Oncomine datasets (Figure 4). The results showed that *MARCH5* mRNA levels were significantly higher in ductal breast cancer, invasive breast cancer, and invasive ductal breast cancer compared with matched normal tissues. Overall, our findings suggest that MARCH5 upregulation is highly associated with breast cancer and that MARCH5 plays an important role in tumor cancer progression. To investigate the association between different types of breast cancer patients and *MARCH5* expression, the breast cancer gene expression miner (bc-Gen-175ExMiner) was used to evaluate the prognostic role of MARCH5 in breast cancer. Both DNA microarray (Figure 5A) and RNA-sequencing data (Figure 5B) confirmed the expression of *MARCH5* mRNA in ER status (ER+ > ER−, *p* < 0.0001) and PR status (PR+ > PR−, *p* < 0.0001). However, the expression of MARCH5 mRNA did not differ significantly in HER2 grade status.

### 3.4. Tissue Microarray Analysis of MARCH5 Protein Expression

**Figure 6:**
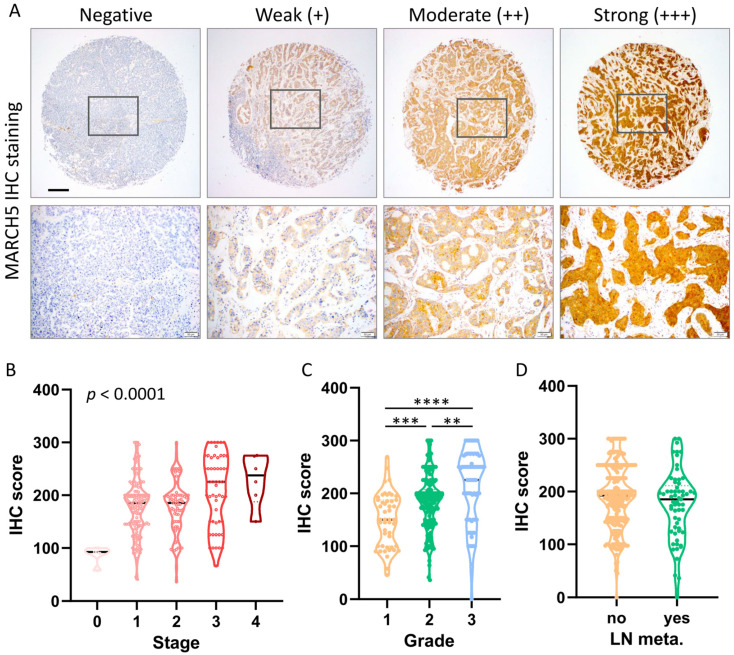
Protein levels of MARCH5 in breast carcinoma. (**A**) Representative images of MARCH5 staining in BRCA tissues. (**B**) IHC scores of MARCH5 expression in BRCA tissues. (**B–D**) Violin plots of MARCH5 expression levels BRCA with significant alterations in different stages, grade, and lymph node metastasis. ** *p* < 0.01, *** *p* < 0.001, and **** *p* < 0.0001. Scale bar = 200 μm.

To further confirm the accuracy of the multi-omics analysis, we evaluated MARCH5 detected using immunohistochemistry in tumor tissues using 60 BRCA commercial tissue microarray (TMA). The results of MARCH5 expression in BRCA tissues in IHC staining are shown in Figure 6A. The IHC score of MARCH5 increased significantly with increasing stage (Figure 6B). At different grades of IHC scores, grade 3 patients showed significantly higher MARCH5 expression than grade 1 and 2 (grade 3 vs. 2, *p* < 0.01; grade 3 vs. 1, *p* < 0.0001, Figure 6C). On the other hand, there was no significant difference in the status of lymph node metastasis (Figure 6D). The results were consistent with the results of the Oncomine database, and MARCH5 expression levels were higher in the late stage.

### 3.5. Co-Expressed Network of MARKCH5 and Correction between Mitochondrial Dynamics Genes in BRCA

**Figure 7:**
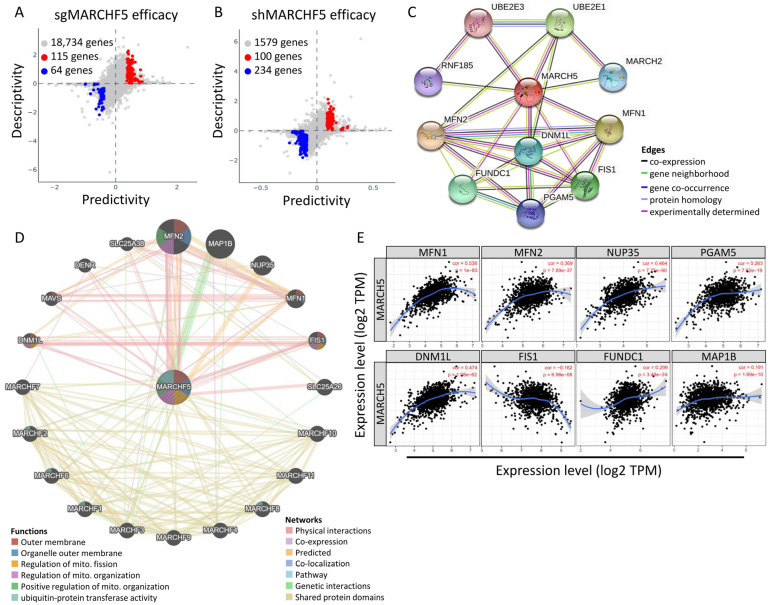
Intergenic correlations, co-expression network, and the biological functions of MARCH5. (**A**,**B**) Bidirectional predictive and descriptive analysis of gene expression in breast cancer cell lines. Predictability and descriptiveness between mRNA expression and sgRNA/shRNA efficacy are plotted. Hit genes showing positive or negative scores with *p*-values <0.01 for both predictive and descriptive aspects were selected. (**C**) PPI network constructed using the STRING database shows MARCH5 and the interacting proteins. The line thickness indicates strength of interaction between any two proteins. (**D**) GeneMANIA database analysis shows that MARCH5 interacts with mitochondrial dynamics proteins. (**E**) The association between *MARCH5* and mitochondrial dynamics-related genes.

To clarify the possible interaction of MARCH5 upregulation with potential genes in breast cancer, we analyzed the predictive and descriptive scores of *MARCH5* compared with potential regulation of mRNA expression by knockout (sgRNA) and knockdown (shRNA) analysis of breast cancer cells for cross-comparison. A total of 115 genes were found to meet the *p*-value < 0.01 criterion for predictive and descriptive scores of mRNA expression and sgRNA validity (red circles in Figure 7A). The hit genes with negative scores (blue circles in Figure 7) showed the smallest predictive and descriptive scores. In addition, a total of 100 genes were likewise found to be predictive of mRNA expression and shRNA validity (Figure 7B). Both predictive and descriptive expression of these genes are important in terms of knockdown and knockout efficiency, although the descriptive scores of the negative hit genes were small when analyzing sgRNA and sRNA. We then constructed a functional protein association network of MARCH5 by the predictive model of molecular action (Figure 7C, STRING datasets), and then constructed a two-layer model to reveal the regulatory network of MARCH5 in BRCA using GeneMania (Figure 7D). In addition to MARCH family proteins, they include proteins associated with MFN2, MAP1B, NUP35, MFN1, FIS1, SLC25A26, DNM1L, FIS1, FUNDC1, and so on. In addition, the correlation between MARCH5 and these potential functional proteins and breast cancer genes was further analyzed. The statistical scatter plot of the database showed that *MARCH5* expression was associated with *MFN1* (Pearson correlation coefficient = 0.538, *p* = 1 × 10^−83^), *MFN2* (Pearson correlation coefficient = 0.369, *p* = 7.89 × 10^−37^), NUP35 (Pearson correlation coefficient = 0.464, *p* = 7.75 × 10^−60^), *PGAM5* (Pearson correlation coefficient = 0.263, *p* = 7.62 × 10^−19^), *DNM1L* (Pearson correlation coefficient = 0.474, *p* = 1.08 × 10^−62^), *FIS1* (Pearson correlation coefficient = 0.464, *p* = 7.75 × 10^−60^) and *RUNX3* (Pearson correlation coefficient = 0.162, *p* = 6.98 × 10^−8^), *FUNDC1* (Pearson correlation coefficient = 0.299, *p* = 3.49 × 10^−64^), and *MAP1B* (Pearson correlation coefficient = 0.191, *p* = 1.69 × 10^−10^) (Figure 7E). These potential co-regulatory proteins were highly and positively correlated with MARCH5; therefore, it was hypothesized that high expression of these proteins was associated with poor prognosis.

### 3.6. MARCH5 Correlated with Immune Infiltration Level in BRCA

**Figure 8:**
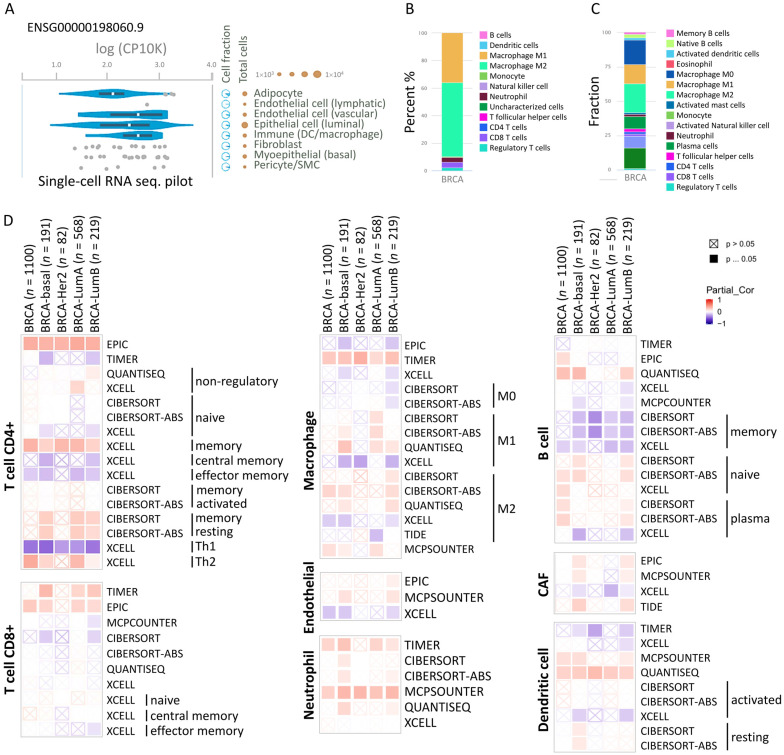
The correlation between *MARCH5* and immunization. (**A**) RNA expression in single-cell clusters of breast cancer by immune marker genes in individual cells by bar graphs. (**B**,**C**) Immune cell percentage and fractionation bars showing the expression of the *MARCH5* gene and well-known cell type markers in different clusters of single-cell types in tissues. (**D**) Heatmap showing the correlation between *MARCH5* expression and immune infiltration in different breast cancer types. Different algorithms explored the potential correlations in *MARCH5* expression level and T cell CD4+, T cell CD8+, macrophage, endothelial, neutrophil, B cell, dendritic cell, and cancer-associated fibroblasts (CAF) across types of BRCA in TCGA.

To clarify the progression of breast cancer and the immune cell response in the tumor microenvironment, we assessed the distribution and type of immune cells in breast cancer tissues using a single-cell RNA-sequencing dataset. We used this database to analyze the potential association of *MARCH5* with the breast cancer tumor microenvironment. We obtained transcriptional data for multiple cell type clusters in this database (Figure 8A). The specificity and distribution classification of these cells were analyzed to determine the differences in the number of genes in these single-cell types and the number of genes detected in all cell types. In Figure 8B, *MARCH5* is shown to regulate different immune cells in breast cancer cells, with macrophage M1 and M2 being the most prevalent among immune cell types. Further fractionation of immune cells revealed that macrophage is the main cluster of cells involved in *MARCH5* regulation of breast cancer. Therefore, we further analyzed the correlation of *MARCH5* with different immune cells through different databases (TIMER). *MARCH5* is involved in immune cell infiltration and inflammatory response and plays a key role in tumorigenesis, progression, and metastasis. Therefore, we used TIMER2, EPIC, MCPCOUNTER.CIBERSORT, CIBERSORT-ABS, QUANTISEQ, XCELL, naive_XCELL, central memory_XCELL, and effector memory_XCELL algorithms to comprehensively explore the correlation between immune cell infiltration and differential expression of *MARCH5* in different cancer types from TCGA. The correlation between immune cell infiltration and differential expression of *MARCH5* from TCGA was explored comprehensively. A positive correlation between MARCH5 expression and macrophage, T cell CD4+, T cell CD8+, and neutrophil was observed in BRCA transitions. (Figure 8D).

**Figure 9:**
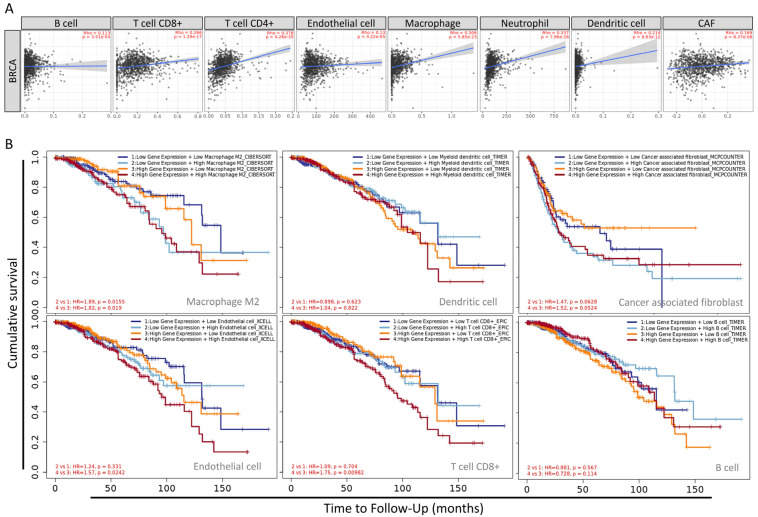
Systematical analysis of immune infiltrates associated with *MARCH5* mRNA expression in BRCA using the Tumor Immune Estimation Resource. (**A**) Correlation of *MARCH5* expression with immune infiltration level in BRCA. (**B**) Kaplan–Meier plots for immune infiltrates and *MARCH5* mRNA expression to visualize survival differences in BRCA.

The statistical scatter plot of the database showed that MARCH5 expression was associated with B cells (Rho = 0.113, *p* = 3.51 × 10^−4^), T cell CD8+ (Rho = 0.266, *p* = 1.29 × 10^−17^), T cell CD4+ (Rho = 0.378, *p* = 4.26 × 10^−35^), endothelial cells (Rho = 0.13, *p* = 4.22 × 10^−5^), macrophage (Rho = 0.306, *p* = 5.85 × 10^−23^), neutrophil (Rho = 0.337, *p* = 7.96 × 10^−28^), dendritic cell (Rho = 0.214, *p* = 8.63 × 10^−12^) CAF (Rho = 0.169, *p* = 8.37 × 10^−8^) (Figure 9A). MARCH5 mediates the migration and localization of immune cells. Increasingly, information suggests that immune cell infiltration can accelerate tumor progression and recurrence, affecting immunotherapy and clinical outcomes. The correlation between MARCH5 expression in BRCA, the abundance of immune infiltrates (macrophages, dendritic cells, CAF, endothelial cells, T cells, and B cells), and survival time is shown in Figure 9A. Patients with high MARCH5 gene expression and high macrophage infiltration all had shorter survival times than those with high gene expression (Figure 9B).

## 4. Discussion

MARCH5 is a mitochondrial ubiquitin ligase regulating mitochondrial fusion/fission-associated protein, such as Drp1 [6], mitofusin 1 [21], and mitofusin 2 [22], which participates in certain phases of cell cycle [21], mitochondria morphology [23], and cellular senescence [24]. Therefore, it is believed that MARCH5 has cancer relevance due to its integral involvement in protein quality control, signal transduction, and cell cycle regulation. According to previous studies, upregulation of *MARCH5* plays a critical oncogenic role in breast carcinogenesis by promotion of both BRCA growth and metastasis [25]. Meanwhile, *MARCH5* expression was significantly higher in epithelial ovarian cancer than in normal ovary tissues, whereas silencing *MARCH5* decreased TGFβ1-induced ovarian carcinomas autophagy, migration, and invasion in vitro and in vivo [26]. In our previous study, we observed that *MARCH5* expression was higher in several cancer types, such as bladder cancer and colorectal cancer, compared with normal tissue. Among them, BRCA expressed the highest *MARCH5* level. We conclude that *MARCH5* provides experimental evidence supporting MARCH5 as a potential therapeutic target in various cancer therapies, especially in BRCA.

Recently, the tumor microenvironment is increasingly recognized as a key player in tumor progression and as a promising therapeutic target in breast cancer [27]. In breast cancer, a high proportion of CD8+ T cells or CD20+ B cells infiltrating the cancer tissue can be a favorable effect on patients’ survival [28,29]. By contrast, several kinds of tumor-infiltrating immune/inflammatory cells, such as Foxp3+ Tregs, CD33+ myeloid-derived suppressor cells (MDSCs), and tumor-associated macrophages (TAMs), were correlated with clinicopathological features, worse breast cancer-specific survival, and shorter disease-free interval [30,31]. TAMs are macrophages that infiltrate tumor tissues or are populated in the tumor microenvironment. Macrophages can be divided into classically activated macrophages (M1 macrophages) and alternatively activated macrophages (M2 macrophages). M1 macrophages possess pro-inflammatory and microbicidal functions, whereas M2 macrophages exert immunosuppression, tumorigenesis, and angiogenesis promotion, as well as tissue reconstruction [32]. In general, TAMs have been shown to express an M2-like phenotype. Few studies have focused on investigating the relationship between *MARCH5* and tumor immune environment. Our previous study first revealed that tumor-infiltrating CD4+ T cell, CD8+ T cell, macrophage, and neutrophil showed a positive correlation with *MARCH5* expression, whereas macrophage M2 is the main cluster of cells involved in MARCH5 regulation of breast cancer. Patients with high *MARCH5* gene expression and high macrophage infiltration all had shorter survival times. Here, we demonstrated the upregulation of *MARCH5* in BRCA tissues through data mining and in vitro experiments, and revealed that *MARCH5* is associated with poor prognosis. In contrast to other studies, this study confirmed the overexpression of *MARCH5* in BRCA, suggesting that MARCH5 could act as a potential oncogene. Otherwise, this study predicted a high positive correlation between *MARCH5* and M2 macrophages by multi-omics. The high expression of *MARCH5* in combination with M2 macrophage resulted in a significant decrease in survival.

Besides M2 macrophages, other immune cells have also been implicated in breast cancer development. A recent study showed tumor-infiltrating CD8+ T cells in breast tumors’ microenvironment were associated with the effect of anti-BRCA immune response, better patient survival, and anti-metastatic progression [29]. Our previous study, however, revealed not only the positive correlation between CD8+ T cells and oncogenic *MARCH5*, but also a decreasing survival when a high expression of *MARCH5* and a high infiltration of CD8+ T cell exist simultaneously. We concluded that tumor microenvironment is multifaceted, often showing different characteristics according to the tumor development and tumor stages. We also suggested that systematic analysis of various tumor-infiltrating immune cells is necessary for providing the findings of the effects of a specific cell combination in immune microenvironment more accurately. 

The present study had some limitations that should be considered. First, only computational, in vitro experiments and patient tests were performed. Further confirmation is needed using specific animal models. Next, only 60 BRCA patients were available for TMA analysis, and future biopsies should be collected consecutively to confirm the association between MARCH5 and immune cells at different times. Genetic mutations and epigenetic and proteomic differences should also be considered in future studies. Finally, despite the multicomponent validation of this study, the molecular mechanisms of the tumor microenvironment remain to be validated.

Mitochondrial morphology is dynamic, and mitochondrial fusion and fission mechanisms regulate these morphological changes that affect mitochondrial function [33]. E2 differentially regulates the transcription of mitochondrial fusion genes, namely MFN1 and MFN2, OPA1 and FIS1, and DNM1L [34]. E2 reduced FIS1 transcripts in MCF-7 cells, but not in MDA-MB-231 or T47D cells associated with increased mtDNA copy number in MCF-7 cells [35]. E2 has an increased expression of NRF1 transcripts and proteins in brown adipose tissue. At this time, females have higher levels of NRF1 transcripts than males [36]. Estrogen regulates mitochondrial function by activating the genomes ERα and Erβ, which stimulate NRF1 transcription, and by directly interacting with mtDNA to promote mtDNA transcription. Estrogen is differentially expressed and integrates cellular metabolism and mitochondrial activity in a cell-specific manner through the estrogen receptor (ER) [36]. Estrogen regulates nuclear gene transcription by binding and activating the classical genomic estrogen receptors α and β (ERα and ERβ). The localization of ERα and ERβ in the mitochondria and mitochondrial membranes provides an additional regulatory mechanism. Although the association of the hormone receptors with MARCH5 has not yet been reported, it is assumed that the hormone receptors regulate mitochondrial dynamics through NRF1 or other transcription factors.

## 5. Conclusions

In this study, we systematically analyzed the expression profile, mutation profile, survival, regulatory network, epigenetics, functional pathways, and immune infiltration associated with *MARCH5* in breast cancer. In addition, we not only found that upregulation of *MARCH5* expression was positively associated with poor prognosis in BRCA, but we also assessed that *MARCH5* transcription is regulated by kinases, mutations, and CNV. Further, *MARCH5* overexpression was found to be involved in mRNA splicing and mitochondrial dynamic imbalance. Finally, MARCH5 may play a role in the immune microenvironment of BRCA. Therefore, MARCH5 may serve as a meaningful diagnostic and sensitive prognostic marker and therapeutic target for immune-related breast cancer. Further studies are needed to confirm these results and to explore the mechanisms and immunomodulatory functions of MARCH5 in BRCA.
